# Mapping the health management journey of women with gestational diabetes mellitus: a qualitative study

**DOI:** 10.1186/s12884-025-08409-y

**Published:** 2025-11-19

**Authors:** Yun Lv, Qianqian Li, Jixia Wu, Xinwei Zhang, Ruting Gu, Yueshuai Pan, Yuting Li, Lili Wei

**Affiliations:** 1 School of Nursing, Qingdao University, Qingdao, 266021 Shandong China; 2 Department of Ophthalmology, The Affiliated Hospital of Qingdao University, Qingdao, 266000 Shandong China; 3 Department of Obstetrics, The Affiliated Hospital of Qingdao University, Qingdao, 266700 Shandong China; 4 Department of Obstetrics, The Affiliated Hospital of Qingdao University, Qingdao, 266100 Shandong China; 5 Department of Thoracic Surgery, The Affiliated Hospital of Qingdao University, Qingdao, 266000 Shandong China; 6 Department of Nursing, The Affiliated Hospital of Qingdao University, Qingdao, 266000 Shandong China; 7 Office of the Dean, The Affiliated Hospital of Qingdao University, Qingdao, 266000 Shandong China

**Keywords:** Gestational diabetes mellitus, Qualitative research, Patient-centred care, Patient journey mapping

## Abstract

**Background:**

Gestational diabetes mellitus (GDM) is a common pregnancy complication that poses challenges to both maternal and fetal health. Effective management requires addressing patients’ varying needs throughout pregnancy and postpartum. This study aims to explore the multi-dimensional health management needs of women with GDM through patient journey mapping, to provide evidence for optimizing integrated care.

**Methods:**

A qualitative study was conducted involving 32 women diagnosed with GDM, recruited via purposive sampling. Data were collected through semi-structured interviews guided by the Patient Safety Systems Engineering Initiative (SEIPS) 3.0 model. Content analysis and patient journey mapping were used to analyse the data.

**Results:**

The health management needs of women with GDM were categorised across tasks, emotions, and pain points according to the diagnosis and treatment timelines. A total of 21 subcategories were identified through content analysis, reflecting key aspects such as emotional distress due to poor glycaemic control, challenges in dietary management, and the influence of family support. These findings were integrated into a comprehensive patient journey map illustrating participants’ evolving needs and experiences across different stages of care.

**Conclusions:**

Patient journey mapping highlights key opportunities to optimise care processes and resource allocation. Enhancing healthcare professional roles and social support, alongside integrating digital health tools, may improve patient experience, self-management, and long-term outcomes.

## Background

Gestational diabetes mellitus (GDM) refers to glucose metabolism abnormalities during pregnancy and is the most common pregnancy complication [1]. According to the International Diabetes Federation (IDF), around 14% of pregnant women worldwide are affected [2]. The global prevalence of gestational diabetes varies significantly across countries and is influenced by differences in screening, diagnostic procedures, and criteria. Furthermore, variations in the underlying population morbidity, along with social, cultural, and risk factors, further contribute to these disparities [3]. In recent years, changes in dietary habits, delayed childbearing, and a greater emphasis on GDM screening have contributed to a dramatic increase in GDM prevalence [4]. The rising incidence of GDM has resulted in significant economic and health burdens globally. Women with GDM are ten times more likely to develop type 2 diabetes postpartum than those without GDM [5, 6]. Furthermore, their offspring are at significantly increased risk of developing metabolic conditions such as obesity and type 2 diabetes.

**Fig. 1:**
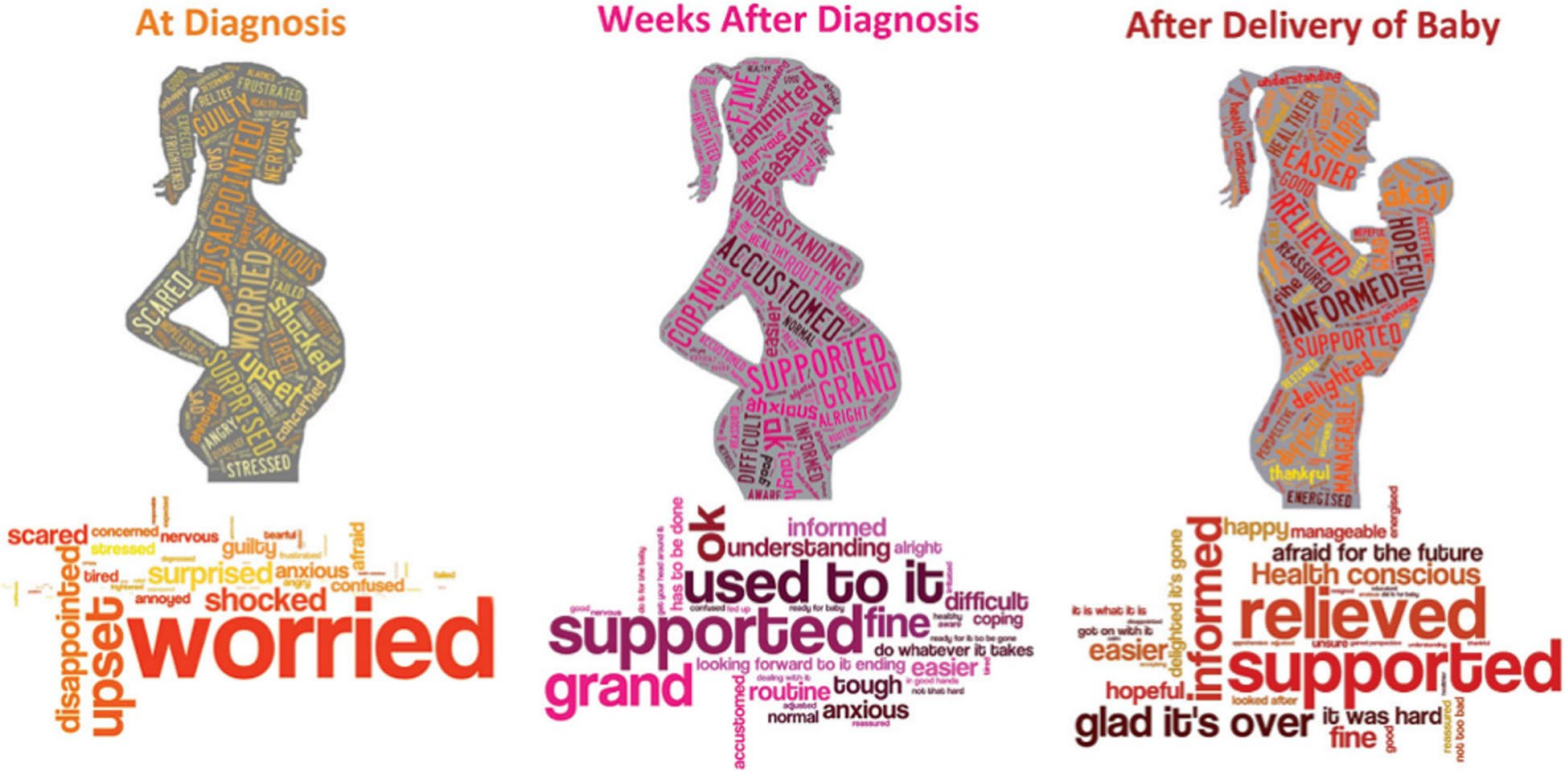
Emotional response to gestational diabetes at different timepoints

While the clinical and economic burdens of GDM are well established, less attention has been given to how women experience care across the continuum of pregnancy and postpartum. Women with GDM encounter multiple challenges, including blood glucose control, physiological and psychological issues, as well as a long and complex management journey. Previous research has shown that women with GDM have an emotional journey after diagnosis (Fig. 1) [7]. The patient journey goes beyond emotional changes. It includes tasks, pain spots, and overall care experience for women from diagnosis until postpartum.

**Fig. 2:**
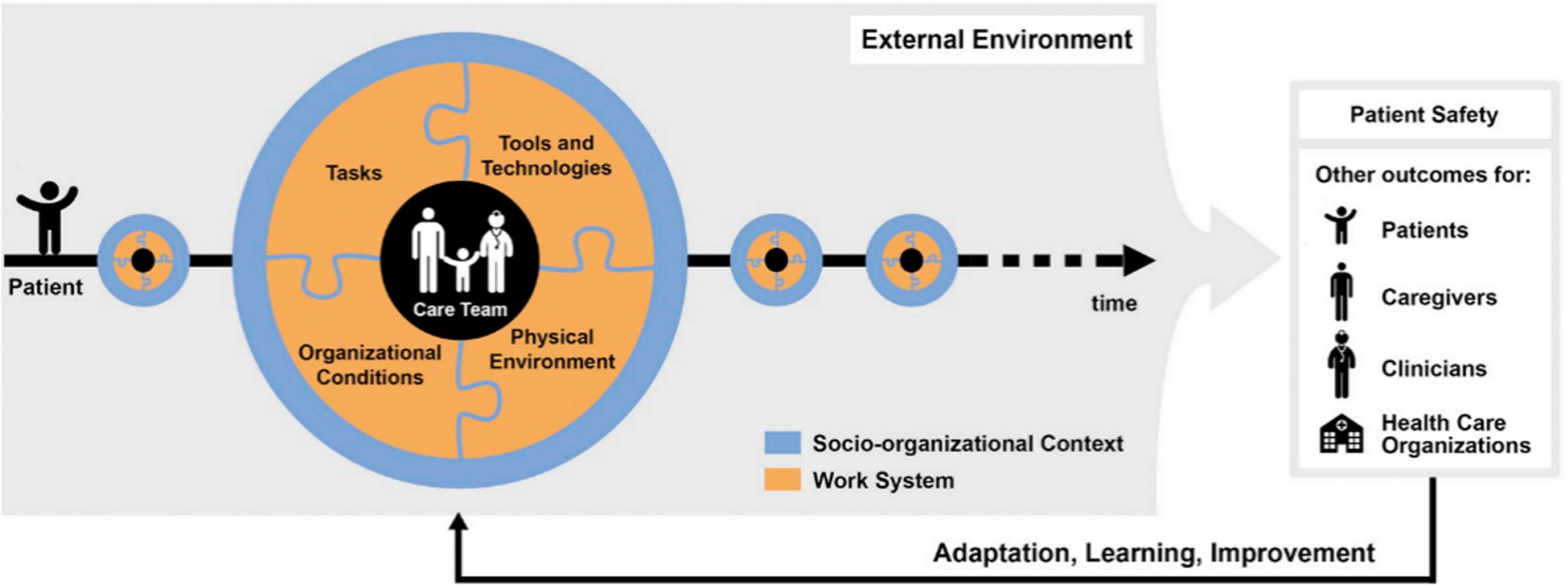
SEIPS 3.0 model: Sociotechnical systems approach to patient journey and patient safety

The term “patient journey” is frequently used in literature and is often regarded as synonymous with “patient pathway” [8]. The patient journey includes not only the care pathway but also the patient’s needs and preferences [9]. It covers the whole process from diagnosis to treatment and ongoing management, including patient experiences and perceptions at each stage, illustrated through a patient journey map [10]. The method focuses on the patient, integrating emotions, behaviours, and needs to offer insights to improve the overall patient care experience [11]. Patient journey maps have recently gained prominence in examining experiences related to cancer and chronic disease management [12, 13]. Such applications highlight the need for a systematic framework to guide the mapping process. The Patient Safety Systems Engineering Initiative 3.0 (SEIPS 3.0) model is regarded as the most comprehensive framework guiding patient journey mapping (Fig. 2) [10]. This illustrates how patients navigate their journeys and interact with six components of the work system: people, tasks, tools and technology, organisational systems, and both internal and external environments at critical touchpoints [14].

Although some studies have explored specific aspects of women’s health management with GDM, comprehensive research on their overall journey remains limited. Most studies on GDM primarily focus on pregnancy or postpartum stages, resulting in limited exploration of the entire disease course. This study aimed to describe and explore the health management experiences, emotional responses, and health needs of women with GDM from screening and diagnosis to postpartum. Given the complexity of women’s experiences with GDM, a qualitative approach was chosen to capture the depth and nuances of their journey, which quantitative methods might not adequately address. In particular, in-depth interviews were used to explore the lived experiences of these women at key touchpoints across the disease course. The SEIPS 3.0 model was used as a framework for the interview guide, helping to structure the exploration of key interactions with various health management components across different stages of GDM. A qualitative descriptive design was employed, and data were analysed using directed content analysis. By constructing a patient journey map for GDM, this study sought to provide a basis for developing more targeted, personalized intervention strategies tailored to the unique needs of women with GDM.

## Methods

### Design

This study adopted a qualitative descriptive design to examine the experiences and needs of women with GDM across the disease trajectory. The aim was to construct a patient journey map informed by these insights. The development of a patient journey map involved conducting desktop research on clinical protocols, utilising observational methods to establish the framework, refining the map through interviews, suggesting improvements, and evaluating it with stakeholders.The interview guide was developed based on a literature review of the six SEIPS 3.0 elements. Interviews included basic demographics and explored experiences and needs across screening/diagnosis, treatment, and postpartum stages. Participants were also encouraged to share additional relevant topics freely. The interview guide was refined through pre-interviews with three healthcare professionals experienced in GDM, and all interviews were conducted by the lead researcher with prior qualitative research training.

### Study setting and recruitment

This study was conducted at a tertiary grade A hospital in Qingdao, China, which is classified as the highest level within the national healthcare system. Participants were recruited through the hospital’s information system, utilising electronic medical records and screening conducted by healthcare personnel. The research team used purposive sampling to gather a broad range of experiences. Upon identifying cases, the researcher explained the study’s purpose and importance to potential participants, face-to-face or by telephone, and obtained consent. Those who agreed were contacted through WeChat, a popular social media platform in China, which is the primary channel for interviews and follow-up communication. Participants could select telephone, WeChat, or face-to-face interviews based on their preferences. Data saturation was reached after interviewing 32 participants. During the study, 43 potential participants were contacted; 13 declined due to health issues or time constraints. Among the 32 participants, 12 were interviewed by phone, 16 through WeChat, and four in person.

### Inclusion and exclusion criteria

The inclusion criteria comprised individuals aged 18 years or older with a history of GDM diagnosed using the American Diabetes Association criteria, who could communicate effectively, were conscious, and could provide informed consent. Participants were interviewed and followed up during pregnancy and up to one year postpartum to reduce recall bias and better understand their experiences and needs at each stage. Among these eligible participants, those with alcohol or other substance dependence, those unable to cooperate due to severe physical or mental illness, or those concurrently participating in other clinical trials were excluded.

### Data collection

Data collection occurred from April 2024 to September 2024. Following a review of the pertinent literature [15] and team consultations, women diagnosed with GDM were interviewed to investigate their experiences and needs throughout the screening, diagnosis, treatment, and postpartum phases. The SEIPS 3.0 model served as the conceptual framework for developing the interview schedule, which captures contextual and system-related factors shaping women’s experiences with GDM. At three stages, the model designs different interview questions. An outline of the interviews is provided in Appendix A.

The demographic data included age, education, occupation, residence, parity, time of diagnosis, and other pregnancy complications. Thirty-two participants were interviewed; no repeated interviews were conducted. The interview lasted approximately 30 minutes. In face-to-face interviews, non-verbal cues were observed to adjust questions accordingly. The recordings were transcribed verbatim into a Word document using professional transcription software within 24 hours. Transcripts were returned to five mothers to check for omissions or errors. Two researchers replayed and reviewed recordings for verification. Demographic data were entered into an Excel spreadsheet.

### Data analysis

In this study, data analysis employed directed content analysis following the approach of Hsieh and Shannon [16]. Each interview was transcribed verbatim by the first author in Chinese to preserve participants’ meaning and nuance. Researchers first conducted preliminary reading of the transcripts and identified meaningful units of text. Second, independent coding and comparisons were performed to finalize the codes. Third, codes were organized and classified according to the pre-established framework of the GDM patient journey (three stages), along with the dimensions of tasks, emotions, and pain points in health management. Fourth, the interrelations among categories and subcategories were examined to refine the category structure. Fifth, recurrent analytic cycles were conducted until no new categories emerged and data saturation was reached. Finally, the journey map framework was integrated with interview data to illustrate changes in participants’ health management needs throughout the journey. These changes were visualized in the patient journey map, and participant representatives reviewed the categories and contributed to refining the map to ensure accuracy and comprehensiveness.

### Ethics approval and consent to participate

Institutional ethical approval was obtained in February 2024 from the institution’s Review Board. The study was conducted in accordance with the ethical principles of the Declaration of Helsinki. All participants provided their written informed consent prior to participation. Consent was obtained electronically via WeChat or other approved digital methods. Participants were informed of the voluntary nature of participation, their right to withdraw at any time prior to data analysis, and the confidentiality measures in place. All identifiable participant information was anonymized, encrypted, and securely stored. Data access was limited to the research team. Clinical trial number: not applicable.

### Rigour and reflexivity

To ensure trustworthiness in accordance with Lincoln and Guba’s criteria [17], several rigorous steps were taken. Credibility was enhanced through peer debriefing and member checking, which confirmed that the findings accurately reflected participants’ perspectives. Dependability was strengthened by having the original Chinese transcripts reviewed by the first author and verified by a second author fluent in Chinese; emerging codes, categories, and subcategories were translated into English, with discrepancies resolved through team discussions. A comprehensive review of final translations and interpretations was performed to maintain consistency and accuracy in coding and analysis. Confirmability was ensured by presenting the final categories and subcategories in both Chinese and English and by including participant quotations to provide context-specific evidence of the health management needs of women with GDM. Transferability was addressed by providing detailed descriptions of participants’ demographics and the research setting, enabling readers to assess the applicability of the findings to similar contexts. Collectively, these procedures strengthened the rigor and trustworthiness of the qualitative descriptive findings.

## Results

### Characteristics of participants

The main characteristics of the participants are summarised in Table 1. The sample predominantly consisted of highly educated women, with 59.4% possessing undergraduate degrees, and healthcare professionals, accounting for 43.8%. The majority resided in urban areas (75%), with a mean age of 34.1 years.

**Table 1:** The participants’ demographic characteristics

Number	Age (years)	Education level	Occupation	Residence	Parity	GDM Dx (wk)^a^	Complications
N1	32	Senior high school	Individuality	County	2	26	No
N2	36	Undergraduate	Education	Urban	1	26	No
N3	37	College	Healthcare personnel	Town	2	26	No
N4	42	Undergraduate	Healthcare personnel	Urban	2	24	No
N5	34	Junior college	Service personnel	Urban	1	26	No
N6	24	Junior high school	Unemployed	Rural	1	20	No
N7	34	Postgraduate	Healthcare personnel	Urban	2	25	Hydronephrosis
N8	33	Undergraduate	Healthcare personnel	County	1	20	No
N9	35	Undergraduate	Healthcare personnel	Urban	2	25	No
N10	41	Senior high school	Worker	County	3	27	No
N11	31	Junior college	Healthcare personnel	Urban	1	24	No
N12	34	Undergraduate	Healthcare personnel	Urban	1	27	No
N13	35	Junior college	Healthcare personnel	Town	2	28	No
N14	33	Undergraduate	Healthcare personnel	Urban	2	24	Hypertension
N15	31	Undergraduate	Healthcare personnel	Urban	2	28	Polycystic ovary
N16	43	Junior college	Worker	County	3	27	No
N17	36	Postgraduate	Healthcare personnel	Urban	2	24	No
N18	42	Postgraduate	Healthcare personnel	Urban	2	24	No
N19	43	Postgraduate	Service industry	Urban	1	18	No
N20	42	Junior college	Finance	Urban	1	28	No
N21	35	Postgraduate	Cargo	Urban	2	24	No
N22	28	Undergraduate	Finance	County	1	20	No
N23	33	Undergraduate	Healthcare personnel	Urban	2	24	No
N24	36	Postgraduate	Healthcare personnel	Urban	2	24	No
N25	41	Senior high school	Cargo	Urban	1	28	No
N26	29	Postgraduate	Teacher	Urban	1	20	No
N27	32	Junior high school	Individuality	Rural	1	25	No
N28	28	Junior college	Individuality	Urban	1	25	No
N29	28	Postgraduate	Engineer	Urban	1	25	No
N30	37	Undergraduate	Teacher	Urban	1	26	No
N31	32	Postgraduate	Educational training	Urban	1	24	No
N32	33	Junior college	Individuality	Town	1	24	No

^a^Gestational diabetes mellitus diagnosis week

### Description of the GDM patient journey

**Fig. 3:**
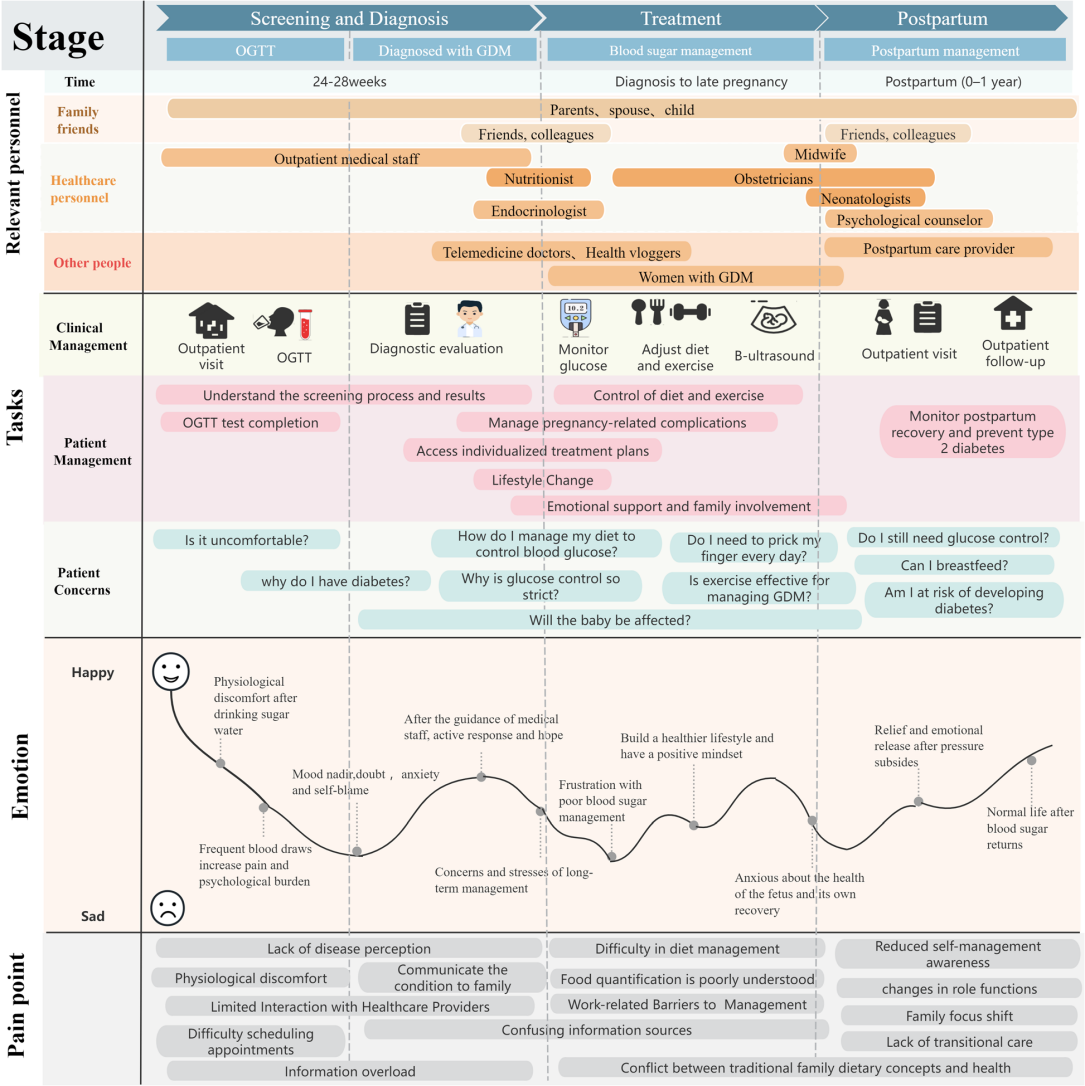
Patient journey mapping in gestational diabetes management

The patient journey of individuals with gestational diabetes was analysed in three stages, focusing on 21 subcategories across three dimensions: health management tasks, emotions, and pain points. The experiences, challenges, and emotional changes of participants during health management were visualised, and their journey across stages and dimensions was systematically mapped (Fig. 3). To enhance conceptual clarity and analytical transparency, Table 2 presents the categories and subcategories identified through content analysis.

**Table 2:** Table of categories and subcategories

No	Stage	Categories	Subcategories
1	Screening and diagnostic stage	Tasks	•Understanding and adhering to screening procedure
			•Engaging in treatment plan decision-making
			•Developing awareness of disease seriousness
		Emotional responses to diagnosis	•Initial psychological resistance
		Pain points	•Physical and procedural burdens
			•Insufficient early education and disease awareness
cline2	Treatment stage	Health management tasks	•Lifestyle-based blood glucose management
			•Understanding blood glucose patterns
			•Family involvement in self-management
		Emotions	•Distress and anxiety over loss of control
			•Validation and enhanced self-efficacy
		Pain points	•Challenges in dietary adherence
			•Contextual barriers to blood glucose management
			•Digital support for self-management
3	Postpartum stage	Tasks	•Continuous blood glucose monitoring
			•Postpartum lifestyle maintenance
			•Managing maternal-infant health balance
		Emotions	•Anxiety about future health
		Pain points	•Decreased attention to self-management
			•Maternal role and self-care conflict
			•Cultural and dietary challenges

### Screening and diagnostic stage

#### Tasks

##### Understanding and adhering to screening procedure

Patients needed to clearly understand the rationale and the strict requirements of the screening process, including fasting, timing, and repeated blood glucose tests. This understanding was essential to avoid errors (e.g., eating during the test, missing steps) that could invalidate results.

*We have to do it once on an empty stomach, then once every hour, and then twice every two hours. It takes a long time. For standard tests, you must check three times, one of which is high, indicating diabetes; therefore, you must check it thoroughly (Participant 5).*.

##### Engaging in treatment plan decision-making

After diagnosis, women’s task was not only to get professional advice but also to decide how to incorporate it into their daily routines. This often meant visiting nutrition clinics and adjusting their diet based on individual recommendations.

*I didn’t know what to do next, so I followed my obstetrician’s advice and went to the nutrition clinic. She said she might be able to help me adjust my diet (Participant 4).*

##### Developing awareness of disease seriousness

Participants must understand the seriousness of gestational diabetes and its potential health risks for both mother and fetus. Gaining a clear understanding of the disease’s importance helps pregnant women see that an accurate diagnosis is not just a routine medical step, but a vital measure to protect maternal and fetal health.

*I must fast according to hospital regulations and arrive on time for the test, as I understand that failing to meet the test requirements could lead to inaccurate results, which would jeopardize the health of both myself and my baby (Participant 7).*

#### Emotional responses to diagnosis

##### Initial psychological resistance

When diagnosed with GDM, participants often initially resist psychologically, showing signs like shock, confusion, or denial. This reaction was especially common among those who saw themselves as healthy, had no previous symptoms, or didn’t think they were at risk. Participants frequently doubted the accuracy of the diagnosis or minimized its seriousness.

*I didn’t understand how I could have it. I don’t even like sweets (Participant 8).*

*I was shocked because everything was normal before 24 weeks, and I did not expect to have diabetes (Participant 14).*

#### Pain points

##### Physical and procedural burdens

Participants described the OGTT as especially difficult, mentioning both the physical discomfort of drinking a concentrated glucose solution on an empty stomach and the hassle of repeated blood draws and long hospital stays. These combined factors made the test stressful and tiring, with several women describing it as a major source of pressure during the screening phase.

*The diagnostic process of drinking sugar water is challenging, not just for me but for many pregnant women in the clinic. Some even vomited shortly after drinking it. The sugar water is hard to swallow; it is super sweet and overwhelming. Moreover, drinking such a high-concentration sugar water on an empty stomach is quite uncomfortable (Participant 18).*

*The frequent blood tests made the process feel overwhelming and forced me to spend much longer in the hospital than I expected (Participant 21).*

##### Insufficient early education and disease awareness

Most participants reported entering the screening and diagnostic stage with little or no prior knowledge of GDM. This “cognitive gap” was mainly linked to the lack of systematic early education and limited public health promotion. Consequently, women’s understanding of GDM often depended almost entirely on information given during their first antenatal visit.

*I feel that the publicity efforts are still insufficient, because you rarely see any promotion about GDM. You only come across relevant educational content when you visit the obstetrics clinic or maternity ward (Participant 15).*

*I am unsure what kind of illness this is because we do not have it in our family, and I do not know anyone around me who has it, so I do not know what it is (Participant 25).*

### Treatment stage

#### Health management tasks

##### Lifestyle-based blood glucose management

Participants mainly relied on lifestyle changes, especially diet and exercise, to control their blood sugar levels. Following recommended meal plans, avoiding foods high in sugar or fat, and engaging in moderate physical activity were common approaches. Many participants described organizing their daily routines around these habits, emphasizing the importance of consistency in keeping blood glucose stable.

*I avoid sweets and fried foods and always take a short walk after lunch and dinner. It’s tiring at times, but I know it helps control my blood sugar (Participant 7).*

*I follow my doctor’s advice on controlling my diet, avoiding fruit as much as possible, and taking a walk after every meal. This has helped me keep my blood sugar levels well under control. (Participant 20).*

##### Understanding blood glucose patterns

Participants highlighted the importance of not only tracking blood glucose but also identifying patterns in their readings and understanding factors that cause fluctuations. This knowledge helped them make better choices about diet, activity, and medication, boosting their confidence in self-management.

*I started noticing my blood sugar was higher in the morning or after certain foods. Knowing this helped me adjust my diet and exercise” (Participant 9).*

*Tracking my readings over several days showed me which habits worked and which didn’t. It gave me more control (Participant 14).*

##### Family involvement in self-management

Participants emphasized the importance of family in supporting blood glucose management by offering oversight and aligning household routines with medical recommendations. Active family involvement improved adherence to lifestyle changes and fostered a shared understanding of disease management.

*Involving my parents during doctor visits helped them understand what I need to do, so they can support me at home(Participant 11).*

*When my husband follows the same diet guidelines, it’s much easier for me to stick to my plan (Participant 22).*

#### Emotions

##### Distress and anxiety over loss of control

When blood glucose levels improve, participants feel a sense of accomplishment and psychological relief, which confirms the effectiveness of their efforts, boosts their self-efficacy, and reinforces their confidence in maintaining a healthy lifestyle.

*Sometimes, even though I’ve been strict with my diet, my blood sugar is still high and hard to control. I’m worried that if I can’t control my blood sugar well, it might affect the health of my baby (Participant 30).*

##### Validation and enhanced self-efficacy

When blood glucose levels improve, participants feel a sense of accomplishment and psychological relief, which affirms the effectiveness of their efforts, boosts their self-efficacy, and reinforces their confidence in maintaining a healthy lifestyle.

*When my blood sugar levels aren’t as high, it feels like all my efforts are paying off, making me happy. It gives me hope that I can have a healthier lifestyle (Participant 31).*

#### Pain points

##### Challenges in dietary adherence

Participants especially struggled with dietary management, finding it difficult to strictly follow dietary restrictions daily and facing challenges with food choices, portion sizes, and practical implementation.

*If you’re trying to measure everything down to the exact grams, it’s hard to do that in a regular household when cooking (Participant 2).*

*The hardest part after getting pregnant is wanting to eat certain things but not daring to, because controlling my diet feels so difficult (Participant 27).*

##### Contextual barriers to blood glucose management

Pregnant women often face time constraints, environmental limitations, and social pressures in their work or daily life, making it difficult to eat at regular intervals or maintain stable blood glucose levels.

*For those of us working during pregnancy, it’s challenging to have snacks in between meals. If we do manage to snack, it’s usually just something like fruit (Participant 2).*

*Because this disease is straightforward, I may be embarrassed to say that I am often hungry, which makes me feel a little hypocritical (Participant 18).*

##### Digital support for self-management

Participants hope to use technological tools, such as apps or digital platforms, to conveniently record their blood glucose levels and dietary intake, receive real-time analysis and personalized guidance, and reduce the need for in-person medical visits.

*It would be great to have a dedicated app that monitors blood sugar levels daily...so you don’t have to go to the doctor every time you measure your blood sugar (Participant 15).*

### Postpartum stage

#### Tasks

##### Continuous blood glucose monitoring

Participants continued to monitor their blood glucose after childbirth due to concerns about developing diabetes. Sustained self-monitoring was seen as essential for early detection and prevention of complications.

*After giving birth, I continue to check my blood sugar regularly to keep track of my health and reduce the risk of developing diabetes (Participant 18).*

##### Postpartum lifestyle maintenance

Some participants successfully integrated the dietary and exercise habits they adopted during pregnancy into a proactive, sustainable approach to managing their health. Even after reaching normal blood glucose levels postpartum, they intentionally limited sugar intake and kept exercising, marking a shift from externally directed management to self-motivated self-care.

*During pregnancy, I learned to control my fruit intake and keep active. Even after giving birth, I still try to limit sugar and engage in regular exercise to maintain my health (Participant 7).*

##### Managing maternal-infant health balance

Participants faced the challenge of balancing their own health management with newborn care. Task conflicts arose when caregiving responsibilities limited the time available for self-care, highlighting the need for strategies that support both maternal and infant well-being.

*After my baby was born, I had to think about both my own health and the baby’s. I try to eat carefully and check my blood sugar when I can, but I have to plan around taking care of the baby (Participant 13).*

#### Emotions

##### Anxiety about future health

After childbirth, participants experienced anxiety related to potential long-term risks of impaired insulin function or developing type 2 diabetes. This concern was compounded by the physical and psychological stress of postpartum recovery, including sleep deprivation and reduced opportunity for exercise.

*I was concerned after giving birth, because some people often say that if this disease is not well managed, it will turn into diabetes (Participant 13).*

#### Pain points

##### Decreased attention to self-management

After postpartum blood glucose levels return to normal, many participants experience a rapid decline in their perceived long-term risk of GDM. Without clear abnormal results as a warning signal, their “patient” identity loses salience, leading to decreased attentiveness in self-management and a diminished sense of urgency to maintain a healthy lifestyle

*“After giving birth, I had my blood sugar tested once and it was normal, so I stopped paying attention to it (Participant 18).*

##### Maternal role and self-care conflict

Postpartum participants face a conflict between caregiving and self-health management. Newborn care demands often lead to deprioritization or interruption of health tasks, while family focus shifts from maternal well-being to infant development.

*Instead, we only focus on whether the child’s development indicators meet the standard. Ordinary families don’t pay much attention to the mother’s situation anymore (Participant 2).*

*After the baby was born, I had no time to think about myself. I stopped checking my blood sugar—I was too tired (Participant 6).*

##### Cultural and dietary challenges

Postpartum women often face a conflict between traditional “confinement” diets and modern medical nutrition advice. Family-endorsed high-calorie diets to support lactation can destabilize blood glucose, while women worry that restrictions may affect milk supply. These cultural beliefs and family pressures make it difficult to follow individualized nutrition plans.

*I didn’t know how to eat it at first, it would be suitable for the milk, and I wouldn’t overgrow, which was after my first child. I have a distorted perspective, and my family does not recognise this aspect of the situation, namely, that the intake exceeds the need. Consequently, I gained 10 pounds during the confinement period, and the amount of milk was not substantial (Participant 17).*

## Discussion

The health management requirements of women with GDM were analysed to guide service design and resource allocation. A patient journey map was used to systematically link the three stages of GDM (screening and diagnosis, treatment, and postpartum) with the dimensions of task, pain point, and emotion, thereby effectively highlighting key categories identified through content analysis. This method combines interview data, clarifies complex needs, and portrays patients’ coping journeys from diagnosis to postpartum across physiological, psychological, and social aspects. Participants face information gaps and emotional distress during screening; experience task overload and role conflict during treatment; and encounter caregiving pressure and reduced self-management postpartum. The research outlined the roles and timing of support personnel, offering a framework for resource coordination and the development of patient-centred, stage-specific support strategies. Recent studies on patient journey maps suggest that data reported by patients can reveal daily needs and emotional shifts, highlighting possible areas for improving patient-centred care [18].

This study employs patient journey map to demonstrate that women with GDM encounter considerable difficulties in regulating blood glucose and managing their health during pregnancy. Screening and diagnosis usually occur during the second trimester; however, dietary habits are generally formed earlier. Participants must acquire knowledge of the disease, monitor blood glucose levels, regulate diet and exercise, and manage emotions during the brief interval between the second and third trimesters to maintain blood glucose within normal limits— a significant challenge. The execution of these tasks greatly impacts maternal and neonatal outcomes, emphasising the vital importance of health education and structured management during the first trimester. With the increasing demands of self-management, digital solutions like blood glucose monitoring applications, telemedicine consultations, and AI-driven health reminders have enhanced glycaemic control and partially mitigated these challenges [19].

Analysis of emotional trajectories indicates that women with GDM experience intricate mood fluctuations, accompanied by persistent concerns regarding severe health outcomes. Participants frequently experience shock and denial during screening and diagnosis, along with distress regarding communication with their families. Previous research has shown that the uncertainty and stress brought by a GDM diagnosis may contribute to anxiety and depressive symptoms, further complicating the early adaptation process [20]. Patients experience anxiety due to repeated finger pricks and uncertainty in monitoring during treatment. This recurring discomfort can sometimes lead to resistance toward blood glucose monitoring, thereby reducing adherence. Previous studies have similarly indicated that the physical burden and psychological stress associated with self-monitoring negatively impact medication adherence [21].Currently, researchers have introduced less invasive alternatives, such as continuous glucose monitoring (CGM) systems utilizing optical sensors, to reduce patient discomfort [22]. During treatment, participants experienced pronounced emotional fluctuations, primarily related to their ability to control blood glucose, concerns about their child’s health, and fears of developing type 2 diabetes in the future. Existing literature has highlighted that women with GDM often face greater psychological stress compared to non-GDM pregnant women, which exacerbates these mood swings [23]. Moreover, studies have shown that emotional fluctuations can, in turn, affect blood glucose levels, creating a bidirectional interaction between psychological well-being and metabolic control [24]. These findings emphasize the importance of psychological support, self-regulation, and stress management strategies during the treatment process to improve adherence and clinical outcomes. It is important to recognise that after childbirth, increased family ignorance and a lack of knowledge about the newborn may contribute to mothers feeling isolated and helpless. Prolonged low mood may lead to postpartum depression. Multiple studies have shown that women with GDM face a higher risk of postpartum depression compared with women without GDM [25]. Therefore, healthcare providers and family should prioritise participants’ psychological well-being and provide support. Encouraging moderate exercise may help participants refocus and regulate blood glucose.

The traditional screening process for Gestational Diabetes Mellitus (GDM), particularly the Oral Glucose Tolerance Test (OGTT), imposes considerable physical and psychological burden, including nausea and vomiting [26]. This burden can contribute to a negative patient experience early in the care journey. Time constraints may limit physicians’ ability to convey comprehensive post-diagnosis information, leading to significant patient anxiety. This anxiety, coupled with unmet information needs, often drives women to seek guidance from non-authoritative sources, potentially compounding distress. To address these systemic challenges, streamlining the diagnostic pathway, such as triaging with Fasting Plasma Glucose to reduce reliance on the full OGTT [27],could alleviate initial burdens. Concurrent with this, healthcare systems should enhance supportive infrastructure. Establishing dedicated health education corners and rest areas within obstetric clinics could provide immediate psychological support and access to reliable information, thereby mitigating anxiety and empowering patients from the point of diagnosis.

Blood glucose management during gestational diabetes mellitus (GDM) treatment is influenced by the complex interplay of multiple factors. Patients commonly report that irregular work schedules, difficulty accessing appropriate meals, or high-intensity work stress in their professional environments impact adherence to prescribed dietary plans. Concurrently, personal factors such as insufficient self-regulation capabilities further exacerbate compliance issues. These challenges highlight gaps in patient education and support systems, including the lack of personalized nutritional guidance and time constraints [28]. Therefore, developing personalized intervention strategies represents a critical step in optimizing blood glucose management. In particular, participants emphasized the need for digital tools to facilitate daily self-care, such as mobile applications that enable convenient recording of glucose levels, provide timely reminders, and deliver tailored dietary guidance. Recent studies have demonstrated that app-based interventions not only enhance self-monitoring and adherence, but also promote more effective communication between patients and healthcare providers.

Postpartum blood glucose management is shaped by both sociocultural and systemic factors. After childbirth, many women shift their attention away from self-care, prioritizing infant care over their own health, while healthcare structures often reduce follow-up for women whose blood glucose levels have returned to normal. Concurrently, adherence to culturally endorsed “high-nourishment” diets aimed at supporting lactation may conflict with individualized medical recommendations [29], further complicating self-management. These combined influences highlight the risk of neglecting maternal health and the potential for suboptimal long-term outcomes. To address these challenges, integrating hospital and community resources is crucial. Linking patient data from hospitals to community health centers allows for structured follow-up, including telephone reminders and digital check-ins via platforms such as WeChat, thereby supporting postpartum women in maintaining healthy lifestyles and reducing the risk of type 2 diabetes [30]. Furthermore, insights from the patient journey mapping highlight the importance of health education and public awareness. Many participants emphasized the need for enhanced promotion of GDM knowledge, suggesting that effective education strategies targeting both patients and the wider community could improve disease awareness, encourage early detection, and support adherence to management plans.

This study provides valuable insights into managing the health of individuals with gestational diabetes, although it has certain limitations. The results demonstrate limited generalizability owing to the qualitative design of the study and the small sample size. Participants were selected from particular geographical and cultural contexts, potentially limiting the generalizability of the findings to all patients’ experiences. In addition, most participants were of older age and relatively high socioeconomic status, with many being well-educated and some working in healthcare, which may not fully reflect the challenges faced by younger women or those from lower socioeconomic backgrounds. Future research should incorporate larger, more diverse samples to explore differences across cultural and socioeconomic contexts.

## Conclusion

By mapping the patient journey for women with gestational diabetes mellitus, this study identified key challenges and unmet needs throughout the screening, treatment, and postpartum stages. The journey map identified key points where patients face emotional distress, struggle with dietary adherence, encounter gaps in follow-up and support, and face other related challenges. These insights provide a foundation for developing targeted, personalized intervention strategies, such as stage-specific education, digital self-management tools, community-based follow-up programs, and enhanced health promotion initiatives. In doing so, healthcare providers can tailor interventions to the unique needs of GDM patients at each stage of their journey, ultimately improving adherence, glycaemic control, and long-term maternal and infant health outcomes.

## Data Availability

The qualitative data generated and analyzed during the current study are not publicly available due to privacy and ethical restrictions, but are available from the corresponding author upon reasonable request.

## Appendix A: Interview outline for health management needs of gestational diabetes based on patient journey maps

Dear expectant/new moms: Our research group is currently doing a project to understand the disease experience and health needs of gestational diabetes patients in different disease journeys (including disease screening and diagnosis stage, post-diagnosis pregnancy stage, and puerperal stage). We want to ask for your assistance in answering the following questions.

Interview content (three stages: screening and diagnosis, treatment, postpartum) Screening and Diagnosis Phases:

1. How did you feel when you learned that you had been diagnosed with gestational diabetes? What do you know about gestational diabetes? (What is gestational diabetes?) How is it diagnosed? How to deal with gestational diabetes? Who did you get information about gestational diabetes from?
2. Did you pay attention to your blood sugar level before you were diagnosed? How did your work and life change and challenge when you were diagnosed with gestational diabetes? What help would you like?
3. Are you satisfied with the tools and techniques used during the screening process? What improvements or new tools do you want to improve screening accuracy and comfort?
4. What do you think of the diagnostic process in hospitals or community organisations (is it reasonable? Are there redundant or repetitive steps?)? What improvements do you think could improve the efficiency and quality of screening?
5. Is regular physical examination a habit in your family? What was your family’s reaction and coping strategy when you were diagnosed with gestational diabetes? What support and help do you want from your family?
6. How do your dietary and social beliefs affect your blood sugar levels at this stage? What support or resources do you hope the government or society will provide to help better screen and diagnose gestational diabetes?

Treatment phase:

1. How do you think the professional ability and service of the treatment team are? What impact has the treatment had on your life? Who have you received help and support from during the treatment?
2. What treatment strategies have you adopted to control blood sugar? What are the difficulties and challenges you encounter in blood sugar management? What are your expectations or suggestions for treatment options?
3. Do you use any tools, apps, or devices to aid in therapy? What new tools or techniques would you like to see help you manage your health more effectively?
4. How do you view the follow-up and management process in existing healthcare facilities or community services? In what areas would you like to improve?
5. How does your home environment affect your blood sugar control at this stage? What blood sugar management support and help would you most like from your family?
6. How do you see social (policy, diet, culture, etc.) support for people with gestational diabetes? In what ways would you like the community to give you more support and help in your treatment process?

Postpartum stage:

1. Do you understand the importance of postpartum blood sugar management? Who do you want to participate in your postpartum blood sugar management?
2. Have you continued to monitor your blood sugar levels after giving birth? What changes have you made in your lifestyle (diet, exercise, etc.)? What tasks do you hope will help you better manage your blood sugar levels?
3. What tools or applications do you use to record and analyse blood sugar data during postpartum blood sugar management? Do you have any suggestions or expectations for postpartum blood sugar management tools?
4. What does the medical system provide for postpartum blood sugar management for you? What other needs do you have?
5. How do you think the internal environment (such as family support, community resources, etc.) affects your postpartum blood sugar management? What improvements and optimisations do you hope to get?
6. At this stage, what are the socio-cultural factors preventing you from managing your blood sugar after giving birth? What improvements would you like to see?
